# Improving the adsorption characteristics and antioxidant activity of oat bran by superfine grinding

**DOI:** 10.1002/fsn3.3054

**Published:** 2022-09-15

**Authors:** Yakun Zhang, Meili Zhang, Xinyue Guo, Xue Bai, Jing Zhang, Rui Huo, YuanYuan Zhang

**Affiliations:** ^1^ College of Food Science and Engineering Inner Mongolia Agricultural University Huhhot P.R. China

**Keywords:** adsorption characteristics, antioxidant properties, functional ingredients, oat bran, physical properties, superfine grinding

## Abstract

Oat bran (OB) is a by‐product of oat, which is rich in β‐glucan. As a new food processing technology, ultrafine powder can improve the surface properties of samples. OB with different grinding times was prepared, and its functional components, physical properties, adsorption properties, and antioxidant properties were evaluated. Results showed that with increased grinding times, the average particle size of OB decreased significantly (*p* < .05). And the water‐holding capacity, swelling capacity, and water solubility index of OB increased significantly (*p* < .05), whereas the animal and vegetable oil‐holding capacities decreased. Oat bran could adsorb cholic acid and glucose, which was related to the time of superfine grinding. In addition, the antioxidant capacity of OB was improved after superfine grinding. Related analysis shows that there was significant positive relationship between β‐glucan, polyphenols and soluble dietary fibers and antioxidant indicators (*p* < .05). The Fourier transform infrared (FTIR) results showed that the FTIR spectra of OB powder with different crushing times were similar. On the basis of the above analyses, it is suggested that OB prepared by superfine grinding for 5 min had good physical and chemical properties and antioxidant properties and is widely used in food.

## Graphical abstract

Ultrafine grinding can improve the antioxidant activity of oat bran (OB). The adsorption capacity of OB can be ameliorated after superfine grinding.

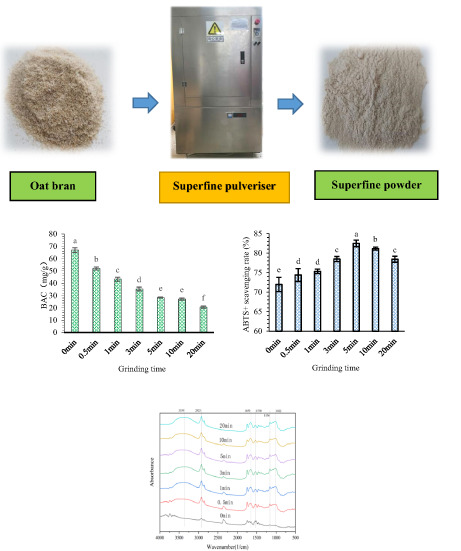

## 1 INTRODUCTION

As a traditional whole grain, oat is considered a healthy food. Oat contains many functional molecules (such as dietary fiber, protein, polypeptide, amino acids, and vitamins) and phytochemicals (such as phytate, lignans, polyphenols, and phenolic acids) (Hu et al., 2014). These substances are concentrated in the outer bran of the grain. Oat bran (OB) is the by‐product of oat and a good source of fiber, vitamins, and phytochemicals. Given that the edible quality of OB is low, OB is often processed to extract the functional components, which are added to food, or simply crushed and added to food. These treatment methods cause environmental pollution and waste of resources.

Excessive cholesterol can cause cardiovascular‐related diseases. Thus, reducing cholesterol intake is important for the prevention and treatment of related diseases (Cofan Pujol, 2014). The biosynthesis of bile acid is the main method of cholesterol catabolism. Reducing the concentration of bile acid in the intestine can accelerate the decomposition of cholesterol in the body, promote the metabolism of cholesterol and reduce the content of cholesterol in the body (Hebanowska, 2010). OB contains a large amount of dietary fiber, which can lower cholesterol and absorb sodium cholate. The intake of OB can also slow down diabetes and prevent and control other chronic diseases, such as cardiovascular disease and obesity (Dong et al., 2016; Thomas et al., 2018; Walters et al., 2020). Besides, OB has good antiliver damage and antioxidant properties (Debnath et al., 2019). These health‐promoting effects are due to the soluble fiber (i.e., β‐glucan) content of oats. In addition to its high dietary fiber content, the phenolic compound contents of OB are high. These phenolic compounds have a high antioxidant capacity and can reduce the risk of chronic diseases (Aaby et al., 2004; Balasundram et al., 2006; Chen et al., 2018). The food industry has an increasing demand for different types of healthy food. Thus, OB is an excellent raw material for functional foods.

However, these chemical substances are preferentially synthesized and accumulated in the outer tissues and cell walls of plant tissues (Zhang et al., 2013) and are difficult to use effectively. Traditional processing methods, such as simply crushed, have difficulty breaking the plant cell wall, which usually leads to the inefficient release of nutrients and functional ingredients. Superfine grinding, a new type of food processing technology, is an effective tool for preparing superfine powders with a particle size of less than 10–25 μm and good surface properties (Hu et al., 2012). This method has been widely used in tea, wheat, and soybeans (Hu et al., 2012; Kong et al., 2020; Zhao, Sun, et al., 2020). However, research on OB is lacking. The particle size of the superfine powder is small and uniform, and the extraction rate of the powder's nutrient and functional ingredients are remarkably improved (Kong et al., 2020). Some studies showed that superfine powder has higher dispersibility, solubility, water retention, antioxidant property, and other important physical and chemical properties (Liu et al., 2016; Zaiter et al., 2016; Zhang et al., 2014). At present, the superfine grinding technology shows remarkable potential in the production of health products and functional foods. Therefore, this experimental system has studied the effects of superfine grinding on the physical and chemical properties of OB.

In this study, the effects of different superfine grinding times on functional components, physical properties, adsorption properties, and antioxidant activity of OB are investigated. This study aims to determine the effects of the grinding times of OB on (1) the content of released bioactive components, such as β‐glucan and phenols; (2) physical properties of OB; (3) adsorption properties of cholesterol, cholate, and glucose, and (4) antioxidation. The results of this study are expected to provide a theoretical basis for the further processing and redevelopment of OB.

## 2 MATERIALS AND METHODS

### 2.1 Chemicals and reagents

The OB was kindly provided by Xibeihuitong Agricultural Technology Co., Ltd.

α‐Amylase, pepsin, and pancreatin were purchased from Sigma‐Aldrich. 1,1‐diphenyl‐2‐picrylhydrazyl (DPPH), 2,2′‐azino‐bis(3‐ethylbenzothiazoline‐6‐sulfonic acid) (ABTS), cholesterol (>99%), bile acid, and *O*‐phthalaldehyde were purchased from Shanghai Macklin Biochemical Co., Ltd. The Megazyme β‐glucan mixed linkage assay kit was purchased from Megazyme International Ireland Ltd.. The total antioxidant capacity (T‐AOC) assay kit was purchased from Nanjing Jiancheng Bioengineering Institute. All other chemicals used in the experiments were of analytical grade.

### 2.2 Sample preparation

The OB (4 W/g) was heated in a microwave oven for 2 min, and powders were ground in a superfine pulverizer (Jinan Pulaishen Machinery Equipment Co., Ltd.) for 0, 0.5, 1, 3, 5, 10, and 20 min. Seven OB powders with different particle sizes were obtained, sealed in bags, and stored in a refrigerator maintained at −20°C.

### 2.3 Determination of main functional components

#### 2.3.1 Total dietary fiber, soluble dietary fiber, and insoluble dietary fiber content

The contents of total dietary fiber (TDF), soluble dietary fiber (SDF), and insoluble dietary fiber (IDF) were determined by enzyme AOAC 985.29 method, and the TDF detection kit of megazyme in Ireland was used.

#### 2.3.2 β‐glucan content

The β‐glucan content was determined using the standard method 32‐23.01 (AACC, 2000) through the Megazyme β‐glucan mixed linkage assay kit (Megazyme International Ireland Ltd.) without ethanol washing.

#### 2.3.3 Total phenolic content

The total phenolic (TP) content was measured in accordance with the method of Dini et al. (2010) with some improvements. The total phenol sample solution was prepared as follows. The sample (1 g) was weighed in a conical flask and added with 60% ethanol in accordance with material to liquid ratio of 1:26. The mixture was extracted in a water bath maintained at 73°C for 62 min, cooled, centrifuged for 20 min at 6000 rpm (Germany Sigma 3‐18k freezing centrifuge; Kaimingjie Instrument Co., Ltd.) and filtered to obtain the supernatant. The total phenol sample solution (1 ml) was added with 1 ml Folin reagent. After sufficient shaking, the solution was allowed to stand for 5 min and added with 2 ml NaCO_3_ (10%) solution. The volume was fixed with distilled water. After 1 h at room temperature, the OD was measured at 765 nm.

### 2.4 Physicochemical properties of OB

#### 2.4.1 Particle size distribution

The particle size was determined in accordance with the method of He et al. (2018). The particle size distribution was measured using the Bettersize 2000 laser particle size analyzer (Dandong Baite Instrument Co., Ltd.). The sample was placed in the container of the particle size analyzer, and the powder was dispersed through ultrasonic wave by using absolute ethanol as dispersant. The particle size and its distribution were determined.

#### 2.4.2 Water‐holding capacity

The water‐holding capacity (WHC) was determined in accordance with the method of Zhang et al. (2017). OB (1.00 g) was accurately weighed (denoted as *m*_1_), placed into a centrifuge tube, centrifuge tube weighing (denoted as *m*_2_), and added with 25 ml deionized water to disperse the powder. The dispersion was stirred at room temperature (20°C ± 3°C) for 4 h and centrifuged at 2500 rpm for 20 min. The supernatant was discarded, and the residual water on the wall of the centrifuge tube was dried with filter paper (denoted as *m*_3_). The WHC was calculated using the following formula: WHC (g/g) = (*m*_3_ − *m*_2_ − *m*_1_)/*m*_1_.

#### 2.4.3 Oil‐holding capacity (saturated and unsaturated fats)

The oil‐holding capacity (OHC) was determined in accordance with the method of Zhang et al. (2017) with certain improvements. About 1 g sample (denoted as *m*_1_) was collected, placed into a centrifuge tube and added with 20 g vegetable oil/lard. The mixture was mixed well, stirred at 37°C for 2 h, and centrifuged at 4000 rpm for 20 min. The upper layer of grease was removed. Free grease was absorbed and weighed (denoted as *m*_2_). OHC was calculated using the formula: OHC (g/g) = (*m*_2_ − *m*_1_)/*m*_1_.

#### 2.4.4 Swelling capacity

The swelling capacity (SC) was determined using the method of Jafari et al. (2017) with certain improvements. Exactly 1.00 g OB (denoted as *m*) was accurately weighed into a test tube with a stopper, and the volume (*V*_1_) was recorded. OB was added with 20 ml distilled water, and the mixture was shaken well and allowed to stand for 12 h at room temperature (25°C ± 3°C). The volume of OB was recorded after absorbing water and swelling (denoted as *V*_2_), and SC was calculated using the formula: SC (ml/g) = (*V*_2_ − *V*_1_)/*m*.

#### 2.4.5 Water solubility index

The water solubility index (WSI) was determined in accordance with the method of Wang et al. (2016). About 1 g OB (*M*_1_) was added into 25 ml distilled water. The solution was transferred into a centrifuge tube, stirred at room temperature (25°C ± 3°C) for 4 h and centrifuged at 2500 rpm for 20 min. The supernatant was poured into a plate and dried to constant weight at 105°C for 5 h. The weight of plate (*M*_2_) and the weight of plate and residue after drying (*M*_3_) were recorded. The WSI was determined using the formula: WSI (g/g) = (*M*_3_ − *M*_2_)/*M*_1_.

### 2.5 Adsorption characteristics of OB in vitro

#### 2.5.1 Sample pretreatment

The digestion experiment in vitro was divided into three parts, i.e., simulating oral cavity, stomach, and small intestine. Kristek et al. (2019) and Mills et al.'s (2008) methods were performed with some modifications. Weigh 20 g OB into a conical flask, add 200 g distilled water, and mix well. In the oral position, 20 mg α‐amylase was dissolved in 6.25 ml CaCl_2_ (1 mm, pH 7.0) and added to the digestate solution, and shaken at 37°C for 30 min. In the stomach position, first adjust the pH to 2.0 with 6 M HCl solution, then dissolve 2.7 g pepsin in 25 ml HCl (0.1 M) and added to the digestive solution, and shaken at 37°C for 2 h. At the small intestine position, the pH was adjusted to 7.0, 560 mg pancreatic lipase and 0.75 g bile were dissolved in 125 ml of NaHCO_3_ (0.5 m), added to the mixture, and shaken at 37° C for 3 h. The digested OB mixture was collected and transferred into a plate and frozen to −80°C. Freeze drying (Beijing Sihuan Scientific Instrument Factory Co., Ltd.) was performed to remove all liquids. Dry samples were collected for subsequent tests.

#### 2.5.2 Bile salt‐adsorbing capacity

The bile salt‐adsorbing capacity (BAC) was determined in accordance with Xu et al.’s method with slight improvement (Fuentes‐Alventosa et al., 2009). The OB digestate sample (0.5 g) was weighed into a 50 ml centrifuge tube and added with 30 ml 0.15 mol/L NaCl solution. The mixture was stirred evenly, shaken in a water bath maintained at 37°C for 2 h, and centrifuged at 4000 rpm for 20 min. Take the supernatant (1 ml) and determine the absorbance at 620 nm by furfural colorimetry. No furfural solution was added into the sample blank group, and no sample solution was added into the furfural blank group. The calculation formula was as follows: Free bile salts content (mg/g) = (free bile salts content in the sample − blank sample − furfural blank)/sample quality.

#### 2.5.3 Glucose dialysis test

The glucose dialysis test was performed in accordance with Fuentes−Alventosa's method (Xu et al., 2015) with some improvements. The OB digestate (0.5 g) was accurately weighed and added with 25 ml distilled water. The mixture was mixed evenly and continuously stirred for 1 h. The sample was transferred into a 15 cm dialysis bag (molecular weight cutoff = 3500 Da), which was placed into a beaker with 200 ml distilled water and shaken at a constant‐temperature shaking incubator maintained at 37°C. At 0.5, 1, 2, 3, 4, and 5 h, 0.5 ml dialysate was collected, and the content of glucose was determined using the phenol–sulfuric acid method.

### 2.6 Antioxidant activity of OB

The antioxidant activity of OB was determined in accordance with Ozkaya et al.'s (2017) method with slight improvements. The sample (3 g) and 30 ml distilled water were placed into a centrifuge tube and mixed evenly to make an OB slurry. The slurry was continuously stirred for 40 min by using a magnetic stirrer. Samples were centrifuged using a high‐speed centrifuge at 6000 rpm for 5 min. The supernatant was collected for the determination of antioxidant properties.

#### 2.6.1 Total antioxidant capacity

The T‐AOC was determined using the Nanjing Jiancheng kit (Nanjing Jiancheng Bioengineering Institute).

#### 2.6.2 DPPH assay

This parameter was determined using the method of Tohma et al. (2017) with some modifications. DPPH· scavenging activity (%) = (1 − [*A*_1_ − *A*_2_]/*A*_0_) × 100%, where *A*_1_, *A*_2_, and *A*_0_ are the absorbance values of the sample, anhydrous ethanol, and blank, respectively.

#### 2.6.3 ABTS assay

The ABTS assay was performed in accordance with the method of Zhu et al. with some modifications (Wang et al., 2009). The ABTS stock solution was prepared as follows. Same amounts of ABTS (7 mM) and potassium persulfate (2.45 mM) were mixed uniformly. The mixture was incubated for 14 h at room temperature under dark conditions. The ABTS working solution was prepared as follows. The ABTS stock solution was diluted with PBS buffer (0.05 M, pH 7.4) until the absorbance at 734 nm was 0.70 ± 0.02. The ABTS^+^ scavenging rate was determined as follows. The working solution (2.85 ml) was mixed with 150 μl sample. The mixture was shaken, allowed to stand for 10 min at room temperature in the dark and centrifuged at 6000 rpm for 5 min. The supernatant was collected, and its absorbance was measured at 734 nm. In addition, the control group with distilled water instead of samples was determined. Zero with distilled water. The ABTS^+^ scavenging rate was determined using the formula: ABTS^+^ scavenging rate (%) = ([OD_cont_ − OD_sam_]/OD_cont_) × 100%, where OD_cont_ and OD_sam_ are the absorbance values of the control and test samples, respectively.

#### 2.6.4 Hydroxyl radical (•OH) scavenging assay

This parameter was determined using the method of Wang et al. with some modifications (Zhu et al., 2011). Ferrous sulfate solution (2 ml, 6.0 mM) and salicylic acid ethanol solution (2 ml, 6.0 mM) were mixed. The mixture was added with 1.5 ml supernatant and 1 ml hydrogen peroxide (8.8 mM), mixed, allowed to stand for 10 min and centrifuged at 6000 rpm for 5 min. The supernatant was collected, and the absorbance was measured at 510 nm. •OH scavenging activity (%) = (1 − [*A*_1_ − *A*_2_]/*A*_0_) × 100%, where *A*_0_: the blank absorbance of the sample is replaced by distilled water, *A*_1_: sample absorbance, *A*_2_: the absorbance of distilled water instead of hydrogen peroxide.

### 2.7 Fourier transform infrared spectroscopy analysis

Refer to the method of Zhang et al. (2021). The appropriate amount of OB was weighed, mixed with KBr, pressed into tablets, and analyzed by infrared spectrometer.

### 2.8 Statistical analysis

All measurements and analyses were performed in three replicates and at least three parallels. Results were expressed as mean ± standard deviation. The one‐way analysis of variance and Duncan multiple comparison test (*p* < .05) were used to determine differences in the physical properties, adsorption, and antioxidant properties of OB under different particle size distributions. Statistical analysis system was used for data analysis.

## 3 RESULTS AND DISCUSSION

### 3.1 Particle size distribution

The changes in particle size and distribution could be used to measure the effect of superfine grinding on powder. *D*[4,3] and *D*[3,2] were the volume‐ and surface‐weighted mean diameters. The values of D10, D50, D75, and D90 showed that 10%, 50%, 75%, and 90% (by volume and quantity) of the sample particles were smaller than this value. For example, D90 means that the size of the whole powder sample was divided into two groups in accordance with the D90 value. About 90% of the particles were smaller than the D90 value, whereas the other 10% of the particles were larger than the D90 value. In addition, D50 was the median of particle size distribution.

Based on the analysis of the particle size distribution of OB powder in Table 1, the values of D10, D50, D75, D90, *D*[4,3], and *D*[3,2] decreased from 194.85, 466.79, 655.39, 852.18, 506.15, and 55.31 μm, respectively, up to 3.32, 11.68, 22.11, 39.6, 17.38, and 7.77 μm, respectively. The D50 values of OB powders indicated that the sample belonged to the superfine food powder grade (Hu et al., 2012). The results of *D*[4,3] showed that the grain size of OB decreased with increased grinding times and decreased significantly at 0–5 min, but no significant difference was observed at 5–20 min. This finding might be due to the agglomeration of single particles after long‐time comminution (Meng et al., 2018). With decreasing particle size, the specific surface area of the sample increased rapidly from 39.83 to 285.88 m^2^/kg, showing an increase of 6.18 times. The increase in the specific surface area might expose active groups, thereby improving the physiological activity of OB (Zhao et al., 2010). In addition, the increase of the specific surface area can significantly improve the taste of the sample, so that it no longer has a rough graininess. Therefore, superfine grinding can remarkably reduce the particle size of powder in food.

**TABLE 1:** Particle size distribution of the oat bran powder

Grinding time (min)	*D*[4,3] (μm)	*D*[3,2] (μm)	D10 (μm)	D50 (μm)	D75 (μm)	D90 (μm)	Specific surface area (m^2^/kg)
0	506.15 ± 11.80^a^	55.31 ± 2.14^a^	194.85 ± 3.53^a^	466.79 ± 32.93^a^	655.39 ± 33.15^a^	852.18 ± 26.55^a^	39.83 ± 1.15^a^
0.5	237.85 ± 2.88^b^	25.81 ± 0.58^b^	8.6 ± 0.10^b^	183.19 ± 14.20^b^	344.2 ± 25.47^b^	549.15 ± 27.18^b^	88.01 ± 2.98^b^
1	52.52 ± 3.50^c^	14.31 ± 0.27^c^	6.53 ± 0.07^b^	28.12 ± 1.32^c^	65.25 ± 4.13^c^	130.04 ± 10.69^c^	155.3 ± 2.91^c^
3	33.95 ± 1.13^d^	10.47 ± 0.31^d^	4.19 ± 0.03^c^	18.9 ± 0.34^c^	43.96 ± 0.83^cd^	90.08 ± 1.63^d^	230.78 ± 1.87^d^
5	24.11 ± 024^e^	9.03 ± 0.17^e^	3.52 ± 0.07^d^	15.22 ± 0.03^c^	32.4 ± 0.45^d^	57.43 ± 1.13^e^	246 ± 4.57^e^
10	23.09 ± 0.45^e^	7.87 ± 0.14^e^	3.18 ± 0.05^d^	12.76 ± 0.10^c^	30.64 ± 0.21^d^	56.35 ± 0.20^e^	282.31 ± 5.11^f^
20	17.38 ± 0.22^e^	7.77 ± 0.01^e^	3.32 ± 0.01^d^	11.68 ± 0.03^c^	22.11 ± 0.21^d^	39.6 ± 0.69^e^	285.88 ± 0.32^f^

*Note*: Values represent means ± standard deviations. Means followed by different letters (a–f) in the same column represent statistically significant differences (*p* < .05).

### 3.2 Effect of superfine grinding on the functional components of OB

The functional components of OB superfine powders with different crushing times are shown in Table 2. Results showed that after superfine grinding, the content of β‐glucan increased first and then decreased with prolonged crushing times, but the difference was not significant (*p* > .05). The contents of β‐glucan in OB with crushing time of 5 and 10 min were 11.59 and 11.63 g/100 g, respectively. After pulverizing for 20 min, the content of β‐glucan suddenly dropped. This result might be because of long crushing time, which destroyed the structure of β‐glucan and reduced the β‐glucan content. With the extension of the superfine grinding time, the total phenol and SDF content also increased first and then decreased, and significant differences among groups were observed (*p* < .05). At a crushing time of 5 min, the TP and SDF content in OB was the highest (579.85 mg/100 g, 16.25 g/100 g, *p* < .05). However, the content of TDF and IDF decreased significantly with the increase of grinding time (*p* < .05).

**TABLE 2:** Effect of superfine powder on the functional components of oat bran.

Grinding time (min)	β‐glucan (g/100 g)	TP (mg/100 g)	TDF (g/100 g)	IDF (g/100 g)	SDF (g/100 g)
0	11.25 ± 0.08^a^	380.40 ± 9.58^f^	27.54 ± 1.06^a^	15.76 ± 0.36^a^	11.94 ± 0.48^d^
0.5	11.37 ± 0.01^a^	409.66 ± 8.46^e^	26.69 ± 0.09^b^	13.39 ± 0.34 ^b^	13.29 ± 0.43^c^
1	11.37 ± 0.14^a^	429.08 ± 2.22^d^	26.09 ± 0.18^c^	12.23 ± 0.18^c^	13.86 ± 0.01^c^
3	11.56 ± 0.10^a^	484.56 ± 10.55^c^	26.04 ± 0.66^c^	11.75 ± 0.12^d^	14.29 ± 0.55^b^
5	11.59 ± 0.02^a^	579.85 ± 10.69^a^	25.64 ± 0.06^d^	9.38 ± 0.56^e^	16.25 ± 0.50^a^
10	11.63 ± 0.04^a^	546.24 ± 15.59^b^	25.45 ± 0.05^d^	9.36 ± 0.40^e^	16.09 ± 0.35^a^
20	11.18 ± 0.33^a^	499.95 ± 10.89^c^	23.24 ± 0.54^e^	8.97 ± 0.07^f^	14.27 ± 0.61^b^

*Note*: Values represent means ± standard deviations. Means followed by different letters (a–f) in the same column represent statistically significant differences (*p* < .05).

Abbreviations: IDF, insoluble dietary fiber; SDF, soluble dietary fiber; TDF, total dietary fiber.

On the one hand, this finding is because the cell wall of OB is destroyed after superfine grinding, and the dissolution resistance of functional components inside the cell is reduced, thereby increasing the dissolution rate of functional components. At the same time, superfine grinding can change and refine the structure of protein and fiber in OB powder and promote the release of bound polyphenols (Das & Eun, 2018). On the other hand, decreasing particle size results in increasing uniformity of powder, gradually increasing contact area between material and solvent and sufficient contact, which accelerates the dissolution rate of polyphenols within a certain period and increases the total phenol content of the micropowder (Cao et al., 2018). If the superfine grinding time is too long, OB is subjected to increased physical forces, resulting in the destruction of the structure of functional components and decreased content (Jiang et al., 2020). The reason for the increase in SDF content is the melting of macromolecular substances, which converts insoluble substances into soluble substances.

A long grinding time does not result in fine powder and high dissolution rate of functional components. For this experiment, the dissolution of functional components can be improved at superfine grinding treatment time of 5 min. Appropriate grinding is conducive to reducing the particle size and increasing the content of bioactive components.

### 3.3 Hydration properties

**FIGURE 1:**
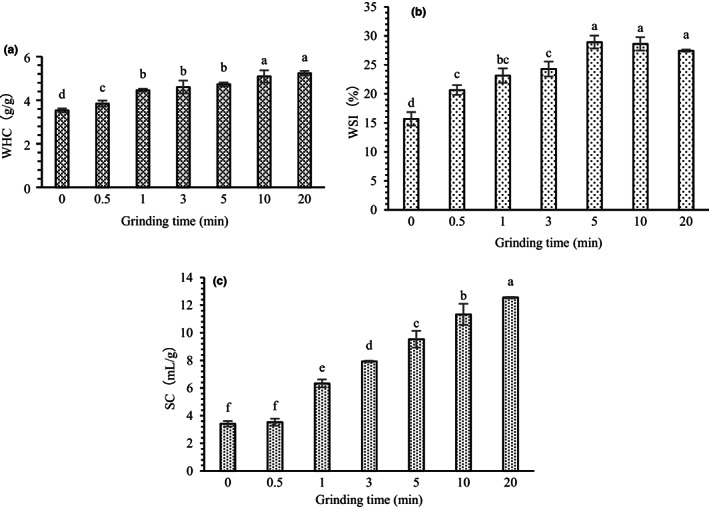
Effects of superfine grinding on the (A) WHC, (B) WSI, and (C) SC of oat bran. Means ± SD (*n* = 3) with different letters (a–f) are significantly different (*p* ≤ .05). SC, swelling capacity; SD, standard deviation; WHC, water‐holding capacity; WSI, water solubility index

Figure 1 shows that with increased grinding time, the WHC and WSI of OB increased significantly (*p* < .05). WHC increased from 3.53 to 5.23 g/g, showing an increase of 48.15%. WSI increased from 3.42 to 12.55 ml/g, showing an increase of 2.67 times. This finding was consistent with the results of previous studies on wheat bran (Zhu et al., 2010). This finding might be because the superfine grinding treatment improves the specific surface area of the sample and increases its contact area with water. The superfine grinding results in altered spatial structure of fibrous substances, increased pores, and easy combination with water (Zhu et al., 2014). The WSI increased first and then decreased. At a crushing time of 5 min, the WSI was the highest at 28.92%, which was 84.44% higher compared with that of the untreated group. This result showed that a small particle size of the sample resulted in increased specific surface area. Thus, increased soluble components can be dissolved, and the water solubility of the sample can be improved.

### 3.4 Oil‐holding capacity

**FIGURE 2:**
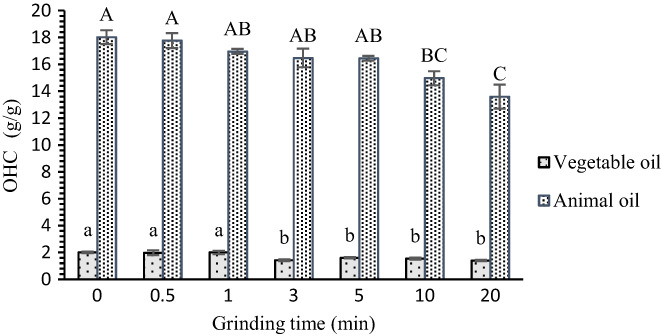
Effect of superfine grinding on the OHC of OB. Lowercase letters (a, b) indicate significant difference in the vegetable OHC of OB at different grinding times (*p* < .05). Capital letters (A–C) indicate significant difference in animal OHC of OB at different grinding times (*p* < .05). No difference analysis between uppercase and lowercase letters was performed. OB, oat bran; OHC, oil‐holding capacity

As shown in Figure 2, the superfine grinding has an opposite effect on the OHC of OB. With increased grinding time, the animal and vegetable OHC of OB decreased. However, OBs subjected to a crushing time of 0–5 min had no significant difference in the ability to hold animal oil (*p* > .05). At grinding time of 20 min, compared with the untreated group, OB exhibited lower OHC values, which were 1.40 (vegetable oil) and 13.60 (animal oil) g/g. This result showed a decrease by 30.40% and 24.53%, respectively. Previous studies found that the OHC of cress (Hua et al., 2019), bagasse (Sangnark & Noomhorm, 2003), wheat bran (Kong et al., 2020), and raw banana (Sangnark & Noomhorm, 2003) show a downward trend after superfine grinding treatment. This finding was consistent with the results of this experiment and might be because of the exposure of increased hydrophilic groups (such as hydroxyl) on the surface of OB and the decreased lipophilicity of OB after superfine grinding. In addition, dietary fiber with porous structure had high affinity for oil. However, after superfine grinding, the content of TDF in the sample decreased, and the structure of dietary fiber was destroyed. The IDF was transformed into SDF (Chau & Huang, 2003). Thus, OHC decreased.

### 3.5 Adsorption characteristics

**FIGURE 3:**
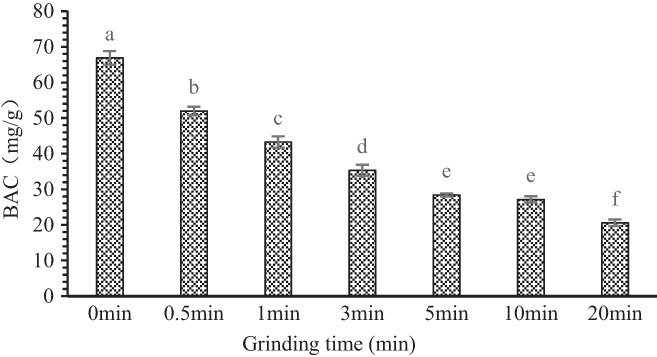
Effect of superfine grinding on the adsorption of bile salts by oat bran. Mean ± SD (*n* = 3) with different letters (a–f) indicate significant differences (*p* ≤ .05). SD, standard deviation

The less free (residual) bile salt content, the better the adsorption capacity of OB to bile salt. Figure 3 shows that OB digestive products had adsorption effect on sodium cholate. With prolonged superfine grinding time, the contents of free sodium cholate, which were 66.92, 52.01, 43.28, 35.34, 28.44, 27.13, and 20.50 mg/g, showed a downward trend. Significant differences were observed among groups (*p* < .05). Therefore, superfine grinding improved the adsorption capacity of OB. After the digestion of OB, most of the remaining substances are dietary fiber. Dietary fiber has a porous structure and has a good adsorption function. Moreover, after the ultrafine pulverization treatment, the specific surface area of the OB powder was increased, and it was easier to form a mucosal layer, which enhances the adsorption capacity. Therefore, the ultrafine pulverization treatment can improve the adsorption capacity of OB digests to bile salts. The BAC of wheat bran was significantly improved after ultrafine grinding (Kong et al., 2020). Most of the bile acids in the human body exist in the form of sodium cholate. OB can effectively absorb sodium cholate in the intestine and excrete it with feces. When the content of sodium cholate decreases, the body will automatically convert cholesterol into sodium cholate for supplementation (Hua et al., 2019). OB reduces the reabsorption of sodium cholate and promotes cholesterol consumption (Bosaeus et al., 1986). Therefore, the higher adsorption capacity of bile acids may help reduce blood lipids and promote human health.

**FIGURE 4:**
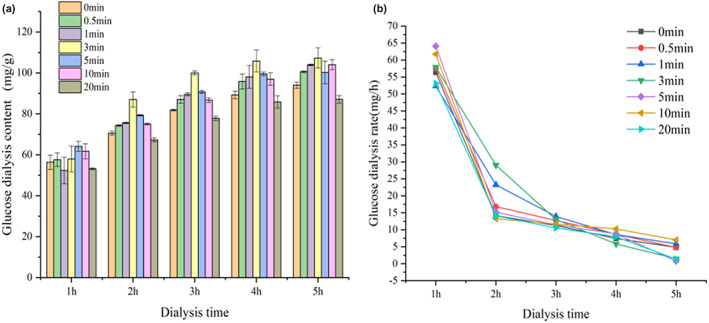
Effect of superfine grinding on (a) glucose dialysis content and (b) dialysis rate of oat bran.

Glucose dialysis is an in vitro indicator of the delayed absorption of glucose in the gastrointestinal tract (Holmes & Mujais, 2006). The effect of superfine pulverization on the delayed glucose absorption capacity of OB is shown in Figure 4a,b. Figure 4a shows that with prolonged dialysis time, the glucose content in the dialysate showed an increasing trend. The 20 min OB had the least amount of glucose dialyzed, while the 3 min OB had the most glucose dialyzed content. After the superfine grinding process, the particle size of the powder becomes smaller, and the starch in the OB was more likely to contact with amylase to produce more glucose, so more glucose may be dialyzed out. However, the amount of glucose dialyzed is related to the structure of dietary fiber. Different types of dietary fiber have different adsorption capacities (Ou et al., 2001). The above research shows that ultrafine pulverization increased the SDF of OB, and its viscosity was higher, which will absorb more glucose (Gao et al., 2020). Therefore, superfine comminution can reduce the amount of glucose dialysis.

With the continuous extension of the dialysis time, the adsorption capacity of the OB digests for glucose was close to saturation, and the adsorption process reached a dynamic equilibrium. The glucose dialysis contents of OB at 5 h followed the order: 3 min >1 min > 10 min >0.5 min >5 min >0 min > 20 min. Gao et al. (2020) found that the asparagus residue subjected to ultrafine pulverization could significantly inhibit the amount of glucose dialyzed. Figure 4b shows that as the dialysis time increased, the glucose dialysis rate showed a downward trend, which slowed down after 3 h. The glucose dialysis rate of OB treated by ultrafine grinding for 20 min was the slowest. After being superfinely pulverized, OB could effectively slow down the diffusion of glucose and provide a theoretical basis for improving postprandial blood sugar.

### 3.6 Antioxidant activity of OB

The excessive accumulation of free radicals destroys the molecules in living cells, thereby endangering human health. Therefore, the timely and effective removal of these free radicals is essential for the body's antioxidant defense (Devasagayam et al., 2004). Antioxidants can protect the human body from free radical damage. These facts have been confirmed in the etiology of many diseases (Xie et al., 2014). The antioxidant activity of OB superfine powder extract determined by four methods is shown in Table 3. As shown in the table, ultrafinely pulverized OB has antioxidant properties and has the highest ABTS^+^ scavenging rate. With increased grinding time, the T‐AOC, DPPH, ABTS^+^, and •OH scavenging rates of OB increased first and then decreased, and significant differences were observed among groups (*p* < .05). At a grinding time of 5 min, the T‐AOC of OB reached the maximum (163.99 U/g OB), and the scavenging rates of DPPH, ABTS^+^, and •OH also reached the maximum (i.e., 68.71%, 82.48%, and 49.38%, respectively).

**TABLE 3:** Effect of superfine grinding on the antioxidant activity of oat bran.

Grinding time (min)	T‐AOC (U/g OB)	DPPH·scavenging rate (%)	ABTS^+^ scavenging rate (%)	•OH scavenging rate (%)
0	65.50 ± 0.69^g^	50.89 ± 1.33^d^	71.98 ± 1.82^e^	34.29 ± 1.89^f^
0.5	71.87 ± 0.92^f^	60.29 ± 1.88^c^	74.39 ± 1.65^d^	35.12 ± 1.17^f^
1	102.57 ± 0.46^e^	63.03 ± 0.59^b^	75.34 ± 0.55^d^	37.74 ± 0.51^e^
3	127.89 ± 0.69^c^	64.08 ± 0.50^b^	78.49 ± 0.65^c^	43.77 ± 1.17^c^
5	163.99 ± 0.92^a^	68.71 ± 0.10^a^	82.48 ± 0.86^a^	49.38 ± 0.51^a^
10	134.59 ± 0.46^b^	68.13 ± 0.51^a^	81.15 ± 0.39^b^	46.60 ± 0.5^b^
20	119.40 ± 0.69^d^	58.42 ± 0.61^c^	78.43 ± 0.78^c^	40.94 ± 0.22^d^

*Note*: Values represent means ± standard deviations. Means followed by different letters (a–g) in the same column represent significant differences (*p* < .05).

Abbreviations: ABTS, 2,2′‐azino‐bis(3‐ethylbenzothiazoline‐6‐sulfonic acid); DPPH, 1,1‐diphenyl‐2‐picrylhydrazyl; T‐AOC, total antioxidant capacity.

Results showed that the antioxidant capacity of OB powder could be improved by superfine grinding. These results were similar to the results of previous studies on the antioxidant capacity of *Lycium ruthenicum* (Zhang et al., 2016) and green tea (Hu et al., 2012) subjected to superfine grinding, which showed an upward trend. In addition, the changing trend of antioxidant activity was consistent with that of functional components. The reason was that after superfine grinding treatment, reduced particle size of the powder, uniform distribution, large specific surface area, and thoroughly broken structure were observed. The more hydroxyl groups of phenolic compounds, the stronger antioxidant activity (Balasundram et al., 2006). Thus, effective antioxidant components were dissolved. β‐glucan and total phenols were the main antioxidant components of OB.

### 3.7 Fourier transform infrared analysis

**FIGURE 5:**
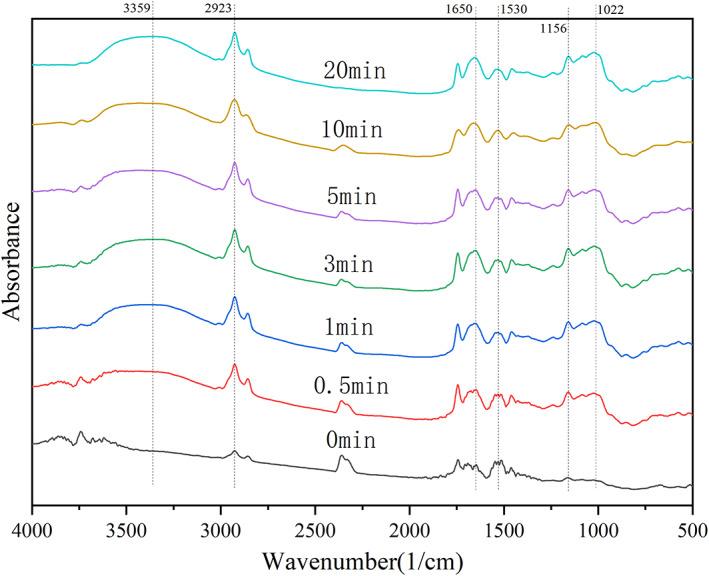
Fourier transform‐infrared analysis of oat bran at different grinding times.

In this study, the molecular characteristics of the powder particles were determined by infrared spectroscopy, and the formation of new groups was detected by spectrogram, so as to determine whether the ultrafine grinding treatment can break the structure of compounds in OB. It can be seen from Figure 5 that the peak position and shape of OB before and after superfine grinding are basically similar, but there are some differences in strength (Zhong et al., 2016). This may be due to the exposure of some groups in OB by superfine grinding or the different scattering intensity caused by different powder sizes. Therefore, the functional groups of OB were not affected and the main components of OB were not changed. The broad peak between 3000 and 3600 cm^−1^ is the stretching vibration peak of O–H in the structure of natural cellulose polysaccharides and polyphenols (Zhao et al., 2018). With the extension of grinding time, the intensity of the peak increased, which was consistent with the change of polyphenol content. The absorption peak between 2800 and 3000 cm^−1^ is the stretching vibration of C–H on –CH2 or –CH3 in hemicellulose polysaccharide (Reddy et al., 2010). The absorption peaks at about 1650 cm^−1^ were C–H on the benzene ring of lignin aromatic compounds, C=O and –COOH stretching vibration in glucuronic acid and polyphenols (Chen et al., 2010). There was an absorption peak at about 1500 cm^−1^, which was the amide II absorption band of secondary amide group, indicating that there was a small amount of protein in OB (Zhao et al., 2015). 1000–1200 cm^−1^ is the contraction vibration of carbohydrate C–O. The peak at 1156 cm^−1^ is caused by the stretching vibration of hemicellulose and cellulose C–O–C. The broad absorption peak at 1022 cm^−1^ is the characteristic absorption peak of C–O–C in hemicellulose sugar ring (Zhao, Meng, et al., 2020). To sum up, OB contains protein, cellulose, hemicellulose, polyphenols, and other substances, and ultrafine grinding does not damage the nutritional components of OB.

### 3.8 Correlation analysis

The correlation between functional components of OB and physical properties and antioxidant activity was shown in Table 4.

**TABLE 4:** Correlation between functional components and physical properties and antioxidation.

Properties		β‐glucan	TP	TDF	IDF	SDF
Adsorption characteristics	BAC	0.293	0.857**	0.820**	0.975**	0.802**
Physical properties	OHC (animal oil)	0.127	−0.512*	0.536*	0.648**	0.531*
	OHC (vegetable oil)	−0.243	−0.727**	0.690**	0.764**	0.592**
	WHC	0.216	0.781**	0.775**	0.917**	0.754**
	WSI	0.414	0.873**	0.698**	0.925**	0.834**
	SC	0.201	0.820**	0.831**	0.933**	0.734**
Antioxidant activity	T‐AOC	0.531*	0.957**	−0.509*	0.854**	0.895**
	DPPH scavenging rates	0.706**	0.806**	−0.25	0.710**	0.886**
	ABTS scavenging rates	0.528*	0.941**	−0.531*	0.902**	0.941**
	•OH scavenging rates	0.615**	0.962**	−0.443*	0.817**	0.902**

Abbreviations: ABTS, 2,2′‐azino‐bis(3‐ethylbenzothiazoline‐6‐sulfonic acid); BAC, bile salt‐adsorbing capacity; DPPH, 1,1‐diphenyl‐2‐picrylhydrazyl; IDF, insoluble dietary fiber; OHC, oil‐holding capacity; SC, swelling capacity; SDF, soluble dietary fiber; T‐AOC, total antioxidant capacity; TDF, total dietary fiber; WHC, water‐holding capacity; WSI, water solubility index.

* Means significant correlation (*p* < .05)

** Mean extremely significant correlation (*p* < 0.01).

β‐Glucan was positively correlated with T‐AOC and ABTS^+^ radical scavenging rate (*p* < .05), and the correlation coefficient was above .5. There was extremely significant positive correlation with DPPH radical scavenging rate and •OH scavenging rates (*p* < .01). There was no significant correlation with other indexes (*p* > .05).

TP and SDF were significantly negatively correlated with OHC (animal oil) (*p* < .05) and extremely significantly negatively correlated with OHC (vegetable oil) (*p* < .01). In addition to OHC, TP and SDF were extremely significant positive correlated with other physical properties, adsorption characteristics, and antioxidant indexes (*p* < .01), and the correlation coefficient between total phenol and antioxidant indexes reached more than .806. The correlation coefficient between SDF and adsorption characteristics was >.802, and the correlation coefficient between SDF and antioxidant index was about .9.

TDF was significantly positively correlated with OHC (animal oil) (*p* < .05) and extremely significantly negatively correlated with other adsorption and physical properties (*p* < .01). There was significant negative correlation with other antioxidant indexes except DPPH radical scavenging rate (*p* < .05).

IDF was extremely significantly positively correlated with OHC (animal oil and vegetable oil) (*p* < .01), and extremely significantly negatively correlated with other adsorption characteristics, physical properties, and antioxidant capacities (*p* < .01).

The contribution value of total antioxidant and ABTS free radical clearance rate was the total phenol > SDF > IDF > TDF β‐glucan.

In summary, the correlation analysis shows that the coefficient between TP, TDF, SDF, IDF and physical characteristics and adsorption characteristics was about 0.7. There was a great correlation between β‐polycagan, TP, SDF, IDF, and antioxidant indicators, and the correlation coefficients are greater than .52; β‐glucan, TP, TDF, SDF, and IDF may be the important functional components of OB that play an antioxidant role.

## 4 CONCLUSIONS

The effects of superfine grinding on the physicochemical properties, adsorption properties, and antioxidant activity of OB were studied. Results showed that the grain size of OB could be effectively reduced and that the physicochemical properties of OB ultrafine powder could be improved by prolonging the grinding time. The OB ultrafine powder obtained by grinding for 5 min had good physical and chemical properties (including WSI; WHC; SC; BAC; CAC; T‐AOC and DPPH‐, ABTS^+^‐, and •OH‐scavenging rates). In addition, more functional components, such as β‐glucan, total phenol, and SDF, were also dissolved. The Fourier transform infrared (FTIR) results showed that the FTIR spectra of OB powder with different crushing times were similar, indicating that the ultrafine crushing did not significantly damage the main structure of the component molecules.

Considering the effects of superfine grinding on the physical properties, adsorption properties, antioxidant properties, and dissolution of characteristic small molecular nutrients of OB, the suitable grinding time of OB was 5 min. The data obtained from the current research provided a theoretical basis for the application of superfine grinding technology in the deep processing of OB and the development of functional food.

## CONFLICT OF INTEREST

The authors declare that they have no conflicts of interest.

## ETHICAL GUIDELINES

Ethics approval was not required for this research.

## Data Availability

Research data are not shared.
